# Supplementary material for: Screening the Impact of Surfactants and Reaction Conditions on the De-Inkability of Different Printing Ink Systems for Plastic Packaging
Source: Polymers (Basel). 2023 May 8;15(9):2220. doi: 10.3390/polym15092220 (PMC10180929; doi:10.3390/polym15092220)
Supplement: Supplementary file 1 [file polymers-15-02220-s001.zip › polymers-2333548-supplementary.pdf]

Screening the De-inkability of Different Surface Printing Ink Systems for Plastic Packaging with Surfactants

Supplement Material S1

1 Structure of the printing binder used in the study

1.1 Nitrocellulose NC

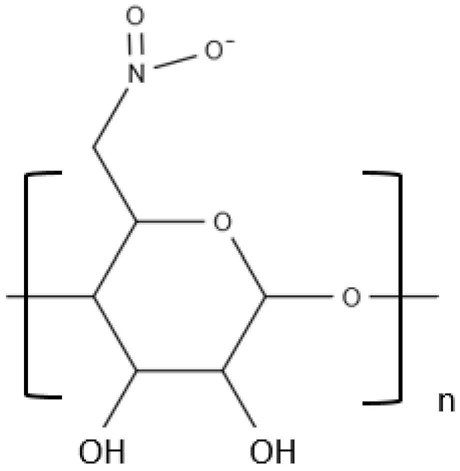

1.2 Polyvinyl butyral (PVB)

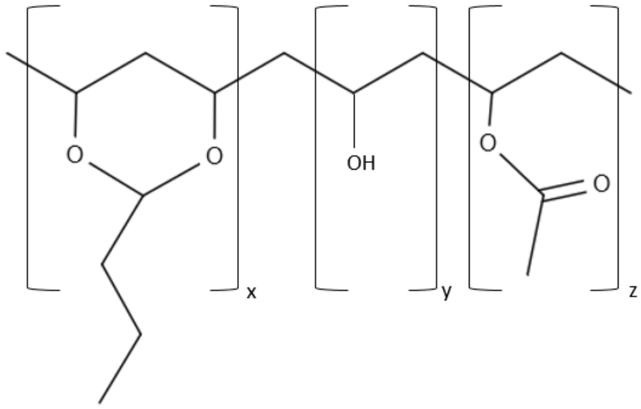

1.3 Cellulose acetate propionate (CAP)

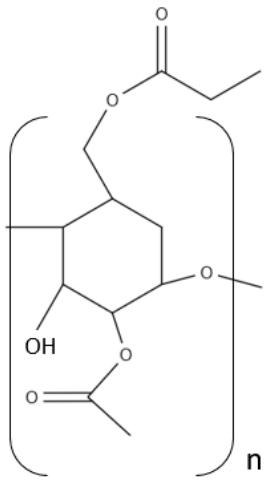

1.4 Polyurethane (PU)

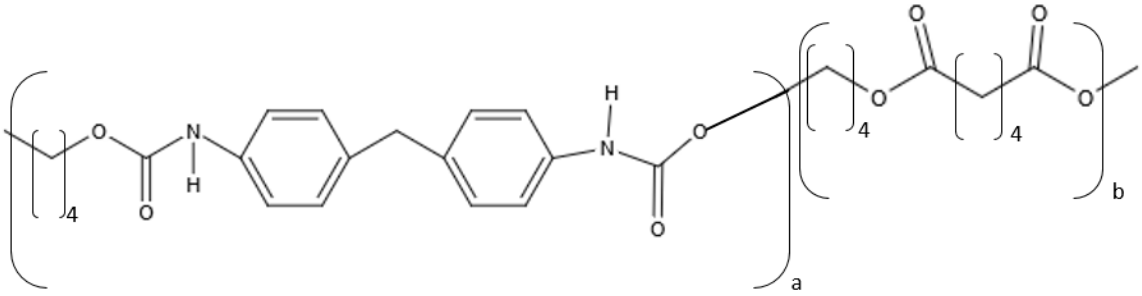

1.5 UV-crosslinked trimethylolpropane triacrylate

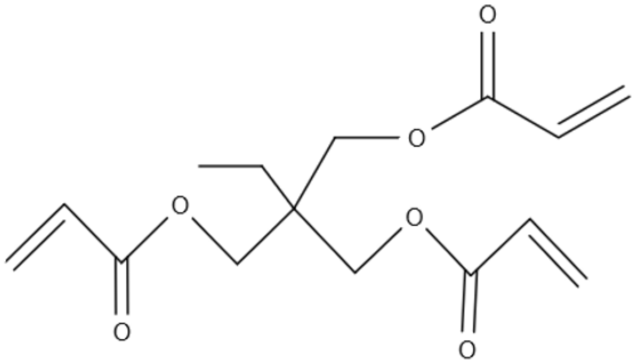

2 Pigment used in the printing inks in this study

Pigment Name	CAS Number
PR 57:1	5281-04-9
PR 146	5280-68-2

PY 13	5102-83-0
PBk 7	1333-86-4
PB 15:4	147-14-8

3 Surfactants used for the de-inking experiment in this study

Commercial name or abbreviation	Full name	CAS number
CTAC	Cetrimonium chloride	112-02-7
W111	Oxirane, methyl-, polymer with 151 oxirane, mono(3,5,5-trimethylhexyl) ether	204336-40-3
CTAB	Cetrimonium bromide	57-09-0
Triton X100	Octoxinol 9	9002-93-1

Supplement Material S2:

Program code for color measurement

```
import gradio as gr

import numpy as np

# from matplotlib import pyplot as plt

from skimage import color



# def lab_preview(img_lab):

#     # scale

#     img_lab_scaled = (img_lab + [0, 128, 128]) / [100, 255, 255]

#     # plot

#     fig = plt.figure()

#     plt.subplots_adjust(hspace=0.25)

#     plt.subplot(221)

#     plt.imshow(img_lab_scaled)

#     plt.title('Lab scaled')

#     plt.subplot(222)

#     plt.imshow(img_lab_scaled[:, :, 0], cmap="gray")

#     plt.axis('off')

#     plt.title('L channel')

#     plt.subplot(223)

#     plt.imshow(img_lab_scaled[:, :, 1], cmap='RdYlGn_r')

#     plt.axis('off')

#     plt.title('a')

#     plt.subplot(224)

#     plt.imshow(img_lab_scaled[:, :, 2], cmap='YlGnBu_r')

#     plt.axis('off')

#     plt.title('b')

#     # convert plot to image using buffer

#     fig.canvas.draw()

#
```

```
#     # Now we can save it to a numpy array.
#     data = np.frombuffer(fig.canvas.tostring_rgb(), dtype=np.uint8)
#     data = data.reshape(fig.canvas.get_width_height()[::-1] + (3,))
#     return data


def lab_mean(img_lab):
    return (
        np.mean(img_lab[:, :, 0]),
        np.mean(img_lab[:, :, 1]),
        np.mean(img_lab[:, :, 2])
    )


def process_lab(img):
    img_lab = color.rgb2lab(img)
    # img_pre = lab_preview(img_lab)
    l, a, b = lab_mean(img_lab)
    return l, a, b


demo = gr.Interface(
    fn=process_lab,
    inputs=gr.Image(),
    outputs=[
        gr.Number(label="L-mean"),
        gr.Number(label="a-mean"),
        gr.Number(label="b-mean"),
        # gr.Image(type="pil"),
    ]
)
```

```python
if __name__ == "__main__":
    demo.launch()
```
